# Perception of arousal in social anxiety: Effects of false feedback during a social interaction

**DOI:** 10.1016/j.jbtep.2006.11.003

**Published:** 2008-06

**Authors:** Jennifer Wild, David M. Clark, Anke Ehlers, Freda McManus

**Affiliations:** Department of Psychology (PO77), Institute of Psychiatry, De Crespigny Park, Denmark Hill, London SE5 8AF, UK

**Keywords:** Social anxiety, Arousal, False feedback, Social behaviour, Social phobia

## Abstract

Cognitive models suggest that during social interactions, socially anxious individuals direct their attention to internal cues of arousal and use this information to erroneously infer how they appear to others. High (*N*=36) and low (*N*=36) socially anxious adults had a conversation with a stooge, and were led to believe by false feedback that they were experiencing either an increase or decrease in arousal, or evaluating the comfort level of the feedback equipment. Compared to the other groups, participants who believed their arousal had increased, reported greater anxiety, poorer perceived performance, more physical cues of anxiety, and greater underestimation of their performance and overestimation of the visibility of their anxiety. The effects were not specific to participants with high social anxiety. Observers rated the behaviour of participants who believed that their arousal had decreased most favourably. The results have implications for the treatment of social phobia.

## 1 Introduction

Social phobia is an isolating anxiety disorder in which sufferers are overly concerned with how they appear to others. They fear saying or doing something that is embarrassing, and this often includes exhibiting physical symptoms, such as blushing, sweating or trembling. They believe other people will notice and then judge them harshly. However, social phobics rarely receive negative feedback in social situations. The persistence of social anxiety despite repeated exposure to their feared stimuli (social situations) and in the absence of direct negative feedback has puzzled clinicians and researchers.

Cognitive models of social anxiety (e.g., Clark & Wells, 1995; Rapee & Heimberg, 1997) conceptualise the attention to, and misinterpretation of, internal information as a key factor in maintaining the disorder. Clark and Wells (1995) propose that when social phobics become concerned about how they are coming across, they shift their attention away from others to detailed monitoring of themselves. The self-monitoring heightens their attention to internal information, which they then use to make erroneous inferences about how they are coming across. The internal information may include somatic information, thoughts and/or images. For example, social phobics may experience a warm feeling in their cheek, believe that it is a sign that they are blushing, believe that blushing is a sign of inadequate social performance, and infer that they are coming across poorly.

A number of studies lend preliminary support to the role of internal cues in social anxiety. Recent research suggests that compared to non-clinical controls, people with social phobia are more likely to interpret physical symptoms, such as blushing, sweating or trembling, as evidencing something negative, such as intense anxiety or a psychiatric condition (e.g., Roth, Antony, & Swinson, 2001). In addition, their fear of exhibiting these symptoms is more likely to heighten their awareness of them. For example, Mulkens, De Jong, Dobbelaar, and Boegels (1999) required high and low fear of blushing individuals to engage in two social tasks which varied in levels of embarrassment. Objective measures of facial coloration and skin temperature indicated that the more embarrassing task produced more coloration for both groups. However, it was just the high fear of blushing individuals who reported greater blushing intensity, indicating a relationship between feared physical symptom of anxiety and heightened awareness of this symptom.

Six studies have provided results consistent with the hypothesis that people with social phobia use internal information to erroneously infer how they appear to others (e.g., Mansell & Clark, 1999; McEwan & Devins, 1983; Mellings & Alden, 2000; Mulkens et al., 1999; Papageourgiou & Wells, 2002; Wells & Papageorgiou, 2001). McEwan and Devins (1983) found that high socially anxious individuals who reported experiencing intense somatic sensations in social situations overestimated how anxious they appeared to their peers. In contrast, there were no discrepancies between self and peer ratings of anxiety visibility for low socially anxious individuals and high socially anxious individuals who reported low-intensity somatic sensations, highlighting a correlation between the perceived intensity and visibility of anxiety symptoms.

Mellings and Alden (2000) required high and low socially anxious individuals to have a conversation with a confederate. Compared to the ratings of an independent assessor, high socially anxious individuals overestimated the visibility of several anxiety-related behaviours and the amount of overestimation was positively correlated with self-focused attention during the interaction.

Mansell and Clark (1999) required high and low socially anxious individuals to give a speech following a social threat induction in which half of the participants were led to believe that their performance was going to be rated by the experimenter and later by a team of psychologists. The other participants were given a no threat induction. Following the speech, participants rated their awareness of bodily sensations during the speech and how well they thought they appeared and performed. An independent assessor also rated participants’ appearance and performance. Both high and low anxious individuals experienced the same incremental increase in anxiety following the social threat induction. There was a significant positive correlation between perceived bodily sensations and the extent to which high socially anxious individuals overestimated the visibility of global negative behaviours (i.e., looking anxious, awkward, unconfident). For both groups, there was a significant positive correlation between perceived bodily sensations and visibility of specific negative behaviours (i.e., sweating, shaking, blushing). The results suggest that high and low socially anxious individuals may use awareness of physical sensations to make judgements about the visibility of specific negative behaviours. But it is the high anxious individuals who go on to use these cues to make negative global inferences about their social performance. The design of this study was correlational, however, so it was not possible to establish a causal role for awareness of physiological sensations, subjective anxiety and perceptions of social performance.

In a more direct attempt to investigate this causal hypothesis, Wells and Papageorgiou (2001) used a false feedback paradigm. Eight patients with social phobia had conversations with a confederate under three different sets of pre-conversation information: (1) no feedback about physiology followed by (2) false feedback of an increased heart rate and (3) false feedback of a decreased heart rate. The latter two conditions were counterbalanced. Patients completed anxiety and belief ratings. Feedback of an increased heart rate led to increments in anxiety, negative beliefs and self-processing. The confederate rated patients as less anxious in the Decrease condition than when they were in the Increase condition. The results suggest that the perception of increased arousal was associated with increased anxiety and actual poorer performance. But did feedback influence actual physiological arousal and was this responsible for increased anxiety?

To investigate this, Papageourgiou and Wells (2002) compared two groups of high and low socially anxious individuals who received information that their heart rate had increased or no information prior to a conversation with a confederate. Actual heart rate was measured throughout the conversation. The results showed that only high socially anxious individuals receiving information about increased heart rate reported significantly greater anxiety and negative social performance. Actual heart rates were not affected by feedback for either high or low socially anxious individuals in either condition, suggesting that body information did not affect actual physiological arousal (as measured by heart rate). Rather, body information influenced subjective anxiety and the perception of visibility of arousal in high socially anxious individuals.

Although these studies lend preliminary support to the Clark and Wells (1995) prediction that social phobics use interoceptive cues during social interactions to interpret how they come across, there are some limitations which warrant further clarification. For example, most studies (e.g., Mansell & Clark, 1999; McEwan & Devins, 1983; Mulkens et al., 1999) have looked at retrospective ratings of physical signs of anxiety rather than online processing of this information and its effect on anxiety and performance. Wells and Papageorgiou (2001) and Papageourgiou and Wells (2002) manipulated the information about the participants’ body states prior to their social interaction rather than during the interaction. Further, participants received only information about their heart rate, which may not correlate directly with other feared physical signs of anxiety that the individual may be concerned about, such as blushing.

The aim of the current study was to assess the effect of online interpretation of internal cues related to physical signs of anxiety on subsequent anxiety and actual and perceived performance. High and low socially anxious individuals were selected for participation. Previous research has indicated the comparability between high socially anxious individuals and those with social phobia (e.g., Mansell & Clark, 1999; Mellings & Alden, 2000). A false feedback paradigm was used in which participants had a conversation with a neutral confederate while receiving information about their level of arousal.

## 2 Method

### 2.1 Overview

High and low socially anxious individuals had a conversation with an experimenter's confederate (who was not aware to which condition the participant had been assigned) while receiving false feedback about their level of arousal, consisting of vibrations delivered to the participants’ chest at set time intervals. The meaning of the vibrations was manipulated experimentally by three different instructions. Participants were told that the vibrations indicated (a) an increase in arousal, (b) a decrease in arousal, or (c) were irrelevant for the experiment (control). We predicted that participants receiving false feedback of increased arousal would (1) show greater subjective anxiety, (2) rate the success of the conversation and their social performance as poorer, (3) report greater perception of bodily sensations, (4) obtain less favourable observer ratings of performance, and (5) underestimate their performance and overestimate the visibility of their anxiety, compared to participants who received false feedback of a decrease in anxiety or control instructions. We further predicted that the differences between the effects of the different instructions would be greater in high socially anxious individuals than in low socially anxious individuals. Finally, we predicted that for participants with high social anxiety, perceived bodily sensations would be associated with low perceived success of the conversation, more negative ratings of their social performance, and greater underestimation of their performance and overestimation of the visibility of their anxiety.

### 2.2 Participants

Participants were 72 university students in London. They were enrolled in a range of disciplines: nursing (*N*=19), architecture (*N*=12), war studies (*N*=10), psychology (*N*=10), biological sciences (*N*=9), art (*N*=5), law (*N*=3), religious studies (*N*=3), and computer science (*N*=1). They were selected because they had scores in the top 25% (17 or greater) and bottom 25% (9 or less) of the general population on the Fear of Negative Evaluation Scale (FNE; Watson & Friend, 1969). Each group had 36 (15 male, 21 female) participants. Participants were randomly allocated to one of three conditions, stratified by sex: Increase, Decrease, and Control, giving a total of six experimental groups. Eighteen additional participants were excluded and replaced: eight because they switched social anxiety category between FNE screening and the experiment, and 10 because they did not believe the experimental manipulation. Of those who switched FNE categories, three were psychology students, and there was one student from each of the following disciplines: history, architecture, nursing, war studies and engineering. Of the ten who did not believe the manipulation, six were nursing students, two were studying architecture, and there was one student studying languages and one studying war studies. There were no significant differences in the proportion of participants with high and low anxiety who believed or did not believe the manipulations. Table 1 shows the mean ages and FNE scores of participants in the high and low social anxiety groups, and their scores on a range of other questionnaires measuring social and general anxiety: the State–Trait Anxiety Inventory, trait and state versions (STAI-T and STAI-S, Spielberger, Gorsuch, Lushene, Vagg, & Jacobs, 1983), the Social Interaction and Anxiety Scale (SIAS, Mattick & Clarke, 1998), and the Autonomic Perception Questionnaire (APQ) (with the instruction to complete it for how they generally feel when they are anxious) (Mandler, Mandler, & Uviller, 1958). As to be expected, *t*-tests indicated significant group differences in all self-report measures, but not in age.

The age of participants and their scores on the questionnaires were submitted to two-way analyses of variance (ANOVA) with two factors: social anxiety and condition. There were no main effects of condition. In addition to the main effects of social anxiety (see Table 1), there were significant interactions of social anxiety and condition for trait anxiety (STAI-T), *F*(2,66)=5.76, *p*<.01, and the APQ, *F*(2,66)=4.61, *p*<.05.

Follow-up ANOVAs with Tukey HSD tests indicated that within the high social anxiety group, participants in the Increase condition scored higher on the STAI-T, *F*(2,33)=5.8, *p*<.01, and APQ, *F*(2,33)=5.1, *p*<.05, than participants in the Decrease and Control conditions, whereas there were no differences on the measures between the conditions for the low anxiety group.

### 2.3 Confederates

There were three female confederates. Two were research assistants who had conversations with the majority of participants (*N*=67). One was a volunteer who had conversations with five of the participants. The experimenter (JW) trained them to act in a pleasant but neutral manner. The instructions were to let the participant initiate and lead the conversation. The confederate could break silences of 10 s or more.

To assess confederate consistency, an independent assessor rated a random selection of video recordings of the confederate's behaviour in the conversations on a 7-point scale, where −3 was unfriendly, 0 was neutral and +3 was friendly. They rated a total of 30 conversations: 10 of the Decrease condition, 10 of the Increase condition and 10 of the Control condition. To assess reliability of the assessor's performance ratings, a psychology research assistant also rated the videotapes of 10 conversations. Inter-rater reliability based on Pearson correlation coefficients was good, *r*=.87, *p*<.001.

### 2.4 Apparatus

The apparatus used to deliver false feedback consisted of a surgical skin pad, mobile phone solenoid, a circuitry control box, and a computer. The surgical skin pad was placed on the back of participants’ necks and was set up to look as though it was measuring heart rate traces. It was attached to a wire that fed to the circuitry control box, which in turn was linked to the computer and the mobile phone solenoid. The solenoid was encased in a small plastic box and taped to the upper right of participants’ chests. The computer delivered nine episodes of vibration to the solenoid via the control box for a duration of 5 s. These were delivered at variable intervals approximately once every minute (i.e., at the following time points during the 8-min conversation: 10, 70, 130, 190, 240, 300, 360, 420 and 478 s).

### 2.5 Instructions

*False feedback*: Participants in the Increase and Decrease conditions were told:

This is the equipment. It monitors your body and gives you feedback during the conversation. It consists of this sensor here and this vibrator. I am going to place this sensor over a blood vessel in the back of your neck. The sensor measures most aspects of arousal. A key component of arousal is heart rate and the sensor picks up any changes in heart rate. The sensor also measures temperature changes of the sort that could happen in blushing. It detects skin moisture allowing it to pick up sweating, and it measures vibrations, allowing it to detect trembling. So, in summary, the sensor measures most aspects of arousal and feeds the information to the vibrator.

Participants in the Increase condition were then told:

If you notice the vibrator going off at the start of the conversation, it's because we’ve definitely picked up an increase in your pulse and level of arousal. If your body continues to have a high pulse rate and level of arousal, the vibrator will tell you this by giving you a signal approximately once every minute.

Participants in the Decrease condition were told:

If you notice the vibrator going off at the start of the conversation, it's because we’ve definitely picked up a decrease in your pulse and level of arousal. If your body continues to have a low pulse rate and low level of arousal, the vibrator will tell you this by giving you a signal approximately once every minute.

The equipment was then attached to the participant and they were shown a fake heart rate trace on the computer to indicate that the equipment was working.

*Control condition*: Participants were told that the piece of equipment had nothing to do with the experiment as follows:

This piece of equipment has actually nothing to do with today's experiment. Instead it's something that we’re developing for a future experiment. And one of the preliminary things we do when we’re creating a new bit of equipment is a basic check on whether it's uncomfortable or not. So, what I’m going to do is at the end of your session today, I’m just going to ask you to tell me whether or not it was uncomfortable while you were wearing it. For the future experiment, the equipment is going to have to vibrate occasionally. And for this reason, our technician may have programmed it to occasionally vibrate while you’re wearing it. If that happens, of course the vibrations have nothing to do with you and have no significance to you. We just want to know whether it was uncomfortable or not at the end of the experiment.

Participants in the Control condition were not shown the fake heart rate trace.

### 2.6 Dependent measures

*Participants’ ratings*: Following the conversation and after the equipment had been removed, participants rated how anxious they had felt during the conversation on a 0–8 scale (‘not at all’ to ‘extremely’). They also rated how well they thought they came across on a 0–8 scale (‘not at all’ to ‘extremely’). This latter question measured their perceived success of the conversation. Participants completed the modified version of Stopa and Clark's (1993) Behaviours Checklist to assess their perception of how they appeared during the conversation (perceived social performance). The questionnaire consists of 15 items: seven global positive descriptors (e.g., confident, friendly, relaxed), four global negative descriptors (e.g., embarrassed, boring), and four specific negative descriptors (e.g., voice quivering, left long gaps in conversation). Each item was rated on a 0–8 scale (‘not at all’ to ‘extremely’). Positive scores were reversed, and the items summed to give a total score indicating the degree to which participants thought they came across badly. The internal consistency for the scale was *α*=.89. Furthermore, participants completed the specific form of the APQ (Bergman & Johnson, 1971; Johansson & Öst, 1982) to assess their perception of bodily sensations during the conversation.

*Confederate's and assessor's ratings of participant's performance*: After the conversation, the confederate completed the Behaviours Checklist—Observer Form (Stopa & Clark, 1993). It included 15 ratings of positive and negative aspects of participants’ behaviours during the conversation. Each item was rated on a 0–8 scale (‘not at all’ to ‘extremely’). Positive scores were reversed and the items summed to give a total score. The internal consistency of the scale was *α*=.89.

The conversation was videotaped and an independent assessor (who was not aware to which condition the participant had been assigned) rated the participant's behaviour on the Behaviour Checklist—Observer Form. The internal consistency of the assessor scale was *α*=.91. To assess reliability of the assessor's performance ratings, a psychology research assistant also rated the videotapes of ten participants’ conversations. Inter-rater reliability for the total score, based on Pearson correlation coefficients, was good, *r*=.90, *p*<.001.

*Underestimation of performance and overestimation of visibility of anxiety*: The extent to which participants underestimated their performance during the conversation was determined by (1) subtracting the confederate's rating on the Behaviours Checklist—Observer Form from the participant's score on the Behaviours Checklist, and (2) calculating a parallel score for the discrepancy from the independent assessor ratings. The extent to which participants overestimated the visibility of their anxiety was determined by subtracting the assessor ratings of how anxious the participant looked from their self-rating of how anxious they thought they looked (on the Behaviours Checklist).

### 2.7 Procedure

Participants were tested individually 1–2 weeks after completing a screening FNE. They had received an information sheet describing the study at screening. The study was explained as a study investigating how different people respond to social interactions. Upon arrival, all participants were reminded that the experiment involved completing some questionnaires and then having a conversation with a stranger while wearing a piece of equipment before completing a final, but brief set of questionnaires. Written informed consent was obtained. Following this, participants completed the following questionnaires: STAI-S, STAI-T, SIAS, FNE, and the APQ. Participants were then taken to another room.

Participants in all three experimental conditions were told that they would have a conversation with a stranger and that they could talk about anything that they wanted to talk about, with the exception of the equipment they were wearing. They received a few suggestions such as where they lived in London, a film they had recently seen, a holiday they had been on or what they were studying. They were fitted with the equipment and received the instructions for the Increase, Decrease or Control condition.

The experimenter (JW) then left the room, and the confederate entered. After 8 min had elapsed, the experimenter returned and informed the participant that he/she could stop talking.

Following the conversation, participants rated how comfortable the equipment was on a 0 (“not at all”) to 8 (“extremely”) scale. They completed the dependent measures and a manipulation check measure. This questionnaire asked them to indicate whether the vibrator had gone off during the conversation, and if so, to estimate how many times it had gone off. Participants in the Increase and Decrease conditions also indicated to what extent they believed the vibrations they felt were giving them information about their bodily sensations on a 0–100 scale (‘not at all’ to ‘completely’). Participants whose belief ratings were below 50% were excluded.

### 2.8 Data analyses

Two-way condition by group ANOVAs tested whether the three experimental conditions (Increase/Decrease/Control) led to differences in self-reported anxiety, perceived performance, perceived physiological symptoms, and observer ratings of the participants’ behaviour for the high and low social anxiety groups. Analyses were repeated including the participant's sex as an additional factor. There were no main effects of sex, with the exception of higher scores for women on the APQ, and no interactions of sex with the experimental factors. To rule out that the pattern of results was due to the observed differences in STAI-T and the APQ, additional two-way analyses of covariance (ANCOVAs) included trait anxiety (STAI-T) and trait APQ scores as covariates with the factors condition and social anxiety group. Significant main effects of condition were further analysed with pairwise ANCOVAs.

Manipulation checks (number of vibrations participants reported, extent to which participants believed the vibrations reflected their bodily responses) were analysed with two-way condition by group ANOVAs. Kruskal–Wallis *χ*^2^ analyses were performed on the independent assessor's ratings of confederate behaviour to assess confederate consistency: in the different conditions and between the different confederates.

Pearson correlations were conducted within each of the social anxiety groups to determine the association between perceived bodily sensations, and the estimation of performance and visibility of anxiety.

## 3 Results

### 3.1 Manipulation checks

The experimental conditions did not differ in the number of vibrations participants reported, and there were no main effects or interactions with social anxiety group (all *p's* >.50). Similarly, there were no differences between the Increase and Decrease conditions in the extent to which they believed that the feedback reflected their bodily state, and no main effects or interactions with social anxiety group (all *p*'s>.80).

There were no differences in confederate behaviour between conditions, using Kruskal-Wallis, *χ*^2^ (2, *N*=30)=2.15, *p*=.342. There were no differences in behaviour between confederates, using Kruskal-Wallis, *χ*^2^ (2, *N*=30)=2.5, *p*=.286.

### 3.2 Participants’ ratings

Table 2 shows the results for the participants’ ratings of their anxiety, perceived success of the conversation, and perceived social performance and bodily sensations during the conversation. The ANOVAs showed highly significant main effects of condition for each of the variables: anxiety, *F*(2,66)=13.78, *p*<.001; success, *F*(2,66)=8.55, *p*<.001; social performance, *F*(2,66)=12.33, *p*<.001; bodily sensations, *F*(2,66)=17.86, *p*<.001. In addition, there were highly significant main effects of social anxiety (all *p*'s<.001), but no interactions. The high social anxiety group scored higher on all measures than the low social anxiety group. When STAI-T and APQ were controlled for by ANCOVA, all condition effects remained significant: anxiety, *F*(2,64)=10.55, *p*<.001; success, *F*(2,64)=6.46, *p*<.005; social performance, *F*(2,64)=9.09, *p*<.001; bodily sensations, *F*(2,64)=15.01, *p*<.001. For all measures, participants in the Increase condition scored higher than those in the Decrease condition (all *p*'s<.005) and those in the Control condition (all *p*'s<.05). Participants in the Decrease condition scored lower than those in the Control condition on the APQ (*p*<.05).

### 3.3 Assessor and confederate ratings of participants’ performance

As shown in Table 2, both the confederate and the independent assessor rated the high social anxiety participants’ performance as poorer than that of the low social anxiety group, *F*(1,55)=19.98, *p*<.001 (confederate) and *F*(1,63)=23.46, *p*<.001 (assessor). There were also main effects of condition, *F*(2,55)=7.34, *p*=.001 and *F*(2,63)=4.32, *p*<.05, respectively, but no interactions. The condition effects remained significant when STAI-T and APQ were controlled in ANCOVAs, *F*(2,53)=6.72, *p*<.005 and *F*(2,61)=3.44, *p*<.05. Participants in the Decrease condition received more favourable ratings than those in the Increase and Control conditions (all *p*'s<.05).

Additional separate analyses of positive and negative items of the Behaviours Checklist showed that the differences between the conditions were mainly due to differences on positive items. For assessor ratings, there were no effects of condition for negative items. For confederate ratings, the ANCOVAs did show a significant condition effect for negative items, *F* (2,53)=3.50, *p*<.05, but a larger condition effect for positive items, *F*(2,53)=7.64, *p*=.001. The item “How anxious did the person look?” did not show effects of condition for either the assessor or the confederate. (All measures showed large effects of social anxiety group, all *p*'s<.001).

### 3.4 Underestimation of performance and overestimation of visibility of anxiety

As shown in Table 2, the high social anxiety group underestimated their performance to a greater extent than the low anxiety group, both compared to the confederate, *F*(1,55)=20.06, *p*<.001, and the independent observer, *F*(1,63)=22.14, *p*<.001, and overrated the visibility of their anxiety, *F*(1,63)=18.17, *p*<.001. There were also main effects of condition, *F*(2,55)=5.65, *p*<.001, *F*(2,63)=6.21, *p*<.005, and *F*(2,63)=11.70, *p*<.001, respectively. The condition effects remained significant when STAI-T and APQ were controlled in ANCOVAs, *F*(2,53)=4.45, *p*<.05, *F*(2,61)=3.88, *p*<.05, and *F*(1,61)=9.10, *p*<.001. Participants in the Increase condition underestimated their performance more than those in the Control condition for confederate ratings (*p*<.05), and, as a trend, for observer ratings (*p*=.064). Participants in the Increase condition overrated the visibility of their anxiety to a greater extent than those in the Decrease (*p* <.01) and Control conditions (*p*<.02*)*.

### 3.5 Correlations

In the high social anxiety group, perceived bodily sensations during the conversation (APQ) correlated with perceived success of the conversation, *r*=−.51, *p*<.005, negativity of perceived performance, *r*=.71, *p*<.001, the degree to which participants underestimated their performance, *r*=.48, *p*<.01, and overestimated the visibility of their anxiety, *r*=.58, *p*<.001. The pattern of correlations was similar in the low social anxiety group, but the size of correlations was lower, *r*=−.32, *p*<.06, *r*=.51, *p*<.001, *r*=.33, *p*=.06, and *r*=.36, *p*<.05, respectively.

## 4 Discussion

In line with the hypotheses, participants who were led to believe that their arousal had increased during a conversation with a stranger felt more anxious, felt that they were coming across badly, and reported more bodily sensations than participants who did not receive feedback on their arousal levels or those who were led to believe that their arousal had decreased. This finding is in line with cognitive models that state that attention to internal cues of perceived arousal in social situations influences anxiety, and actual and perceived performance.

However, contrary to expectation, we did not find that this effect was more pronounced in participants in high compared to low social anxiety. False feedback of increased arousal affected participants with low social anxiety to the same extent as those with high anxiety, although their overall level of anxiety was lower and they rated their social performance during the conversation more favourably. The finding of similar increments in anxiety in high and low anxiety groups is consistent with Mansell and Clark (1999) who found that both groups experienced the same increments and level of anxiety following a social threat induction. It is possible that information regarding increased arousal acts in the same way as perceived social threat possibly drawing attention to the importance of performance and immediate attention on the self. However, if this were solely the case, then one would expect no differences in anxiety and performance between high and low socially anxious individuals in the false feedback study of Papageourgiou and Wells (2002). They found that only high anxious individuals receiving information about increased heart rate reported significantly greater anxiety and negative performance. The effect was not seen in low anxious individuals. However, they provided information about heart rate only, rather than physiological information which could correlate with a wide range of anxiety symptoms and social performance concerns, such as blushing, shaking, and sweating. Further, false feedback was provided prior to participants’ conversations. It was therefore not possible to determine the online impact of such information. Low anxious individuals may not attend to body state information throughout a conversation when this information is provided beforehand. However, when it is provided online, they may be more likely to shift their attention and attend to it with a subsequent impact on anxiety and performance.

The most likely reason for the pattern of results then is that the experimental instructions focused the attention of all participants’ on their “arousal”, regardless of social anxiety status. There was no difference between the high and low social anxiety groups in the number of vibrations they detected or in the extent to which they believed the feedback reflected their bodily state. Thus, low socially anxious participants in the Increase condition were experimentally made to behave like patients with social phobia, in that they were made to shift their attention away from the conversation to the monitoring of their increasing arousal. This would explain why they showed greater anxiety and thought they came across less favourably than low socially anxious people in the Decrease and Control conditions. Other research suggests that when people are able to allocate their attention freely, high socially anxious people are more likely than those with low social anxiety to shift their attention away from facial expressions to internal cues in conditions of social-evaluative threat (Mansell, Clark, & Ehlers, 2003), and would therefore be expected to be more likely to experience the adverse effects of monitoring their increasing arousal.

This fits with the Clark and Wells (1995) cognitive model of social phobia in which self-focused attention is a key maintaining factor. Self-focused attention facilitates self-monitoring and hence, attention to physical cues of arousal while at the same time preventing individuals with the disorder from processing how they are actually coming across. Wells and Papageorgiou (2001) found that information about increased arousal was associated with increased self-focused attention in patients with social phobia. They suggest that attention may be the mechanism by which perception of arousal affects anxiety. Self-focused attention has been associated with anxious appearance (e.g., Woody, 1996) and poorer performance (e.g., Daly, Vangelisti, & Lawrence, 1989).

In this study, high socially anxious participants overrated how negatively they came across to a greater extent than low socially anxious participants, compared to both the confederate and the independent assessor. This is in line with Mellings and Alden (2000) who found that socially phobic and non-anxious individuals overestimated their anxiety-related behaviour relative to observers, but socially phobic individuals did so to a greater extent. Furthermore, as expected, the degree of overestimation varied with the experimental condition. Participants who thought that their arousal had increased showed greater overestimation than participants in the Control condition.

Did participants actually use their perceived physiological state to infer how they came across to others? The effects of the experimental manipulation on participants’ ratings of their social performance, and the pattern of correlations support this relationship. In the high social anxiety group, perceived bodily sensations during the conversation correlated highly with the perceived success of the conversation and more negative ratings of social performance. In addition, perceived bodily sensations correlated with the degree of underestimation of performance and overestimation of anxiety visibility, in line with the findings of Mansell and Clark (1999) and McEwan and Devins (1983).

The experimental manipulation not only affected the participants’ impression of how they came across, but also the actual impression they made on the confederate and an independent assessor. In line with previous studies of social phobia, the high social anxiety group made a less favourable impression overall (Alden & Wallace, 1995; Jones & Carpenter, 1986; Norton & Hope, 2001; Stopa & Clark, 1993). As expected and consistent with Wells and Papageorgiou (2001), participants in the Increase condition made a less favourable impression than participants in the Decrease condition. Further, participants in the Decrease condition were also rated more positively than participants in the Control condition. However, there was no significant difference between the Increase and Control conditions, in contrast to the self-report measures.

The most likely explanation for the overall pattern of findings is as follows: Believing one's arousal is increasing during a social interaction has a large impact on the individual's perception of how well the interaction is going and how well they come across, as these are influenced by anxiety level and perceived bodily sensations. However, this internal state is not necessarily obvious to the social interaction partner and other observers, as indicated by the absence of condition effects on the item “How anxious did the person look?”, and on negative items of the Behaviours Checklist for the independent assessor. Other people appear more likely to pick up differences in positive behaviours, and accordingly, the effects of the experimental manipulation on the assessor ratings were restricted to positive behaviours. The positive behaviours rated by the observers favoured the Decrease group, which had been reassured by the feedback that their arousal was decreasing and may thus have come across in a more relaxed and accessible way. Thus, the pattern of results suggests, in line with models of social phobia (Clark & Wells, 1995; Rapee & Heimberg, 1997), that relying on internal anxiety cues to infer how one comes across does not give a good estimate of what others perceive during a social interaction.

The present results support several procedures used in cognitive therapy (Clark et al., 2003) to treat social phobia. Discussion and behavioural experiments (including videofeedback) are used to help patients to realise that focusing their attention on their anxiety symptoms gives a misleading impression of how they come across and makes them feel more anxious. They are also taught to shift their attention from monitoring their internal state to the interaction with others, which seems to be helpful in both correcting their impression that they are performing badly and in creating a more favourable impression on others.

The present study had some limitations. First, the sample size was relatively small and the random allocation led to a difference in trait anxiety and trait APQ scores between high social anxiety participants allocated to the Increase versus Decrease and Control conditions. However, the pattern of this difference does not explain the pattern of results observed for the dependent measures (main effects of condition). Furthermore, ANCOVAs established that the differences between the experimental conditions remained significant when STAI-T and APQ scores were controlled statistically. Second, the experiment depended on false feedback and thus relied on the extent to which participants believed the manipulation. A proportion of participants who did not believe the manipulation had to be excluded. The majority of them were nursing students who may have had more knowledge of physiology than students of other disciplines. Although we did not observe systematic differences between people who did and did not believe the feedback, it is conceivable that the results do not generalise to all people. Third, the participants were student volunteers selected on the basis of their scores on a measure of social anxiety, and it remains to be tested whether they generalise to patients with social phobia. However, the results of previous studies using other paradigms suggest that such a generalisation is likely (Chen, Ehlers, Clark, & Mansell, 2002; Mansell, Clark, Ehlers, & Chen, 1999; Wells & Papageorgiou, 2001, 2002). Finally, we assessed bodily sensations during the conversation by self-report and did not measure actual changes in physiology. Thus, we are unable to determine whether the instructions led to any group differences in the participants’ arousal. It is unlikely given that Papageourgiou and Wells (2002) found no differences in actual physiological arousal between high and low socially anxious individuals when they were given false feedback of increased arousal before a conversation with a confederate. Other studies have also found no difference in actual heart-rate during a social task between socially anxious and non-socially anxious individuals (e.g., Grossman, Wilhelm, Kawachi, & Sparrow, 2001; Puigcerver, Martinez-Selva, Garcia-Sanchez, & Gomez-Amor, 1989).

This study looked at online false feedback of arousal and found that participants who were given false feedback of increased arousal during a conversation reported greater anxiety, poorer perceived performance, more physical cues of anxiety, and greater underestimation of their performance and overestimation of the visibility of their anxiety. Although the effects of false feedback were similar across high and low social anxiety groups, the feedback may have further influenced those with high social anxiety in that their baseline anxiety is greater. Inducing attention to increased internal sensations demonstrates how socially anxious individuals may be at a further disadvantage in social situations. Shifting attention away from the negative meaning of internal cues of arousal (as in the Decrease condition) improved performance and anxiety. This supports techniques used in cognitive therapy of social phobia and underscores the need to help sufferers shift attention away from internal cues of arousal.

## Figures and Tables

**Table 1:** Participant characteristics

	Low social anxiety (*N*=36)		High social anxiety (*N*=36)		*t*-test
	*M*	SD	*M*	SD	
Age	24.3	5.2	22.0	5.0	*t*(1,70)=1.89, *p*>.05
FNE	4.7	2.5	23.9	3.7	*t*(1,61.19)=25.51, *p*<.001
STAI-S	29.4	7.2	44.9	10.0	*t*(1,70)=7.62, *p*<.001
STAI-T	31.4	7.3	51.4	9.7	*t*(1,70)=9.96, *p*<.001
SIAS	9.9	5.9	34.9	14.2	*t*(1,46.7)=9.78, *p*<.001
APQ	31.2	14.3	52.4	16.5	*t*(1,70)=5.85, *p*<.001

**Table 2:** Effects of the experimental manipulation on anxiety, perceived performance, and observer ratings of the participants’ behaviour

Means (SD)	High social anxiety			Low social anxiety		
	Increase (*N*=11)	Decrease (*N*=12)	Control (*N*=13)	Increase (*N*=13)	Decrease (*N*=12)	Control (*N* =11)
*Participants’ ratings*						
Anxiety (0–8)	6.0 (1.3)	3.0 (1.6)	4.5 (2.1)	2.7 (.9)	1.4 (.8)	1.6 (1.4)
Perceived success of conversation (0–8)	3.0 (2.4)	5.3 (1.4)	4.8 (1.6)	5.1 (1.3)	6.3 (.6)	6.1 (1.8)
Social performance (BCh)⁎	77.1 (15.2)	48.8 (14.9)	55.8 (24.9)	42.4 (17.3)	26.4 (9.1)	27.4 (11.4)
Perceived bodily sensations during conversation (APQ)	44.5 (12.1)	17.4 (8.1)	31.0 (16.7)	23.9 (10.6)	11.6 (7.5)	15.0 (10.6)
*Observer ratings of participants’ performance*						
Independent assessor (BCh)⁎	44.5 (14.4)	35.9 (10.6)	41.7 (8.6)	32.3 (9.0)	24.0 (8.2)	30.6 (8.1)
Confederate (BCh)⁎	40.6 (9.2)	31.5 (13.3)	40.4 (11.7)	27.5 (13.0)	13.5 (6.0)	30.3 (14.3)
*Participants’ underestimation of their performance*						
Compared to independent assessor	32.6 (15.4)	13.3 (10.5)	17.2 (18.5)	10.2 (20.1)	4.0 (11.7)	−3.3 (11.5)
Compared to confederate	40.0 (12.1)	18.5 (15.6)	21.7 (19.2)	13.7 (19.7)	13.0 (8.6)	−2.9 (16.9)
Visibility of anxiety	4.3 (2.1)	1.5 (2.3)	2.8 (2.1)	2.5 (1.6)	.2 (1.3)	.5 (1.0)

APQ: Autonomic Perceptions Questionnaire; BCh: Behaviours Checklist.

⁎ Low scores indicate favourable ratings.
